# The Investigation of Nfκb Inhibitors to Block Cell Proliferation in OSCC Cells Lines

**DOI:** 10.2174/0109298673309489240816063313

**Published:** 2024-08-26

**Authors:** Yuxi Cheng, Liping Wang, Shuju Zhang, Wei Jian, Bin Zeng, Long Liang, Zhiyuan Deng

**Affiliations:** 1 Hunan Key Laboratory of Oral Health Research & Hunan 3D Printing Engineering Research Center of Oral Care & Hunan Clinical Research Center of Oral Major Diseases and Oral Health & Academician Workstation for Oral-maxilofacial and Regenerative Medicine & Xiangya Stomatological Hospital & Xiangya School of Stomatology, Central South University, Xiangya Road, Changsha, 410011, China;; 2 Hunan Children's Hospital, Changsha, 410011, China;; 3 Dental Department, Yueyang Central Hospital, 39# Dongmaoling Road, Yueyang, 414000, China;; 4 Department of Stomatology, Changsha Medical University, Changsha, Hunan, 410219, China;; 5 Hunan Province Key Laboratory of Basic and Applied Hematology, School of Life Sciences, Molecular Biology Research Center, 110# Xiangya Road, Changsha, 410011, China

**Keywords:** Oral squamous cell carcinoma (OSCC), NFκB, LY2409881, MLN4924, inhibitor, neddylation

## Abstract

**Background:**

Oral cancers, with oral squamous cell carcinoma (OSCC) as the predominant type, have a significant impact on morbidity and mortality rates. Therefore, targeting the NFκB pathway shows promise in cancer therapy.

**Materials and Methods:**

This study investigated the impact of two NFκB inhibitors, LY2409881 and MLN4924, on cell proliferation, apoptosis susceptibility, and *in vivo* tumorigenesis in OSCC cell lines CAL27 and SCC15.

**Results:**

The results revealed that both LY2409881 and MLN4924 effectively suppressed cell proliferation, induced apoptosis, and arrested the cell cycle at the G2/M phase—a phenomenon likely associated with the NFκB pathway. Furthermore, MLN4924 demonstrated potent inhibitory effects on cell proliferation at low μM concentrations, surpassing the effectiveness of LY2409881 as an inhibitor (All results: *p*<0.05).

**Conclusion:**

These findings highlight the potential of LY2409881 and MLN4924 as novel therapeutic agents for OSCC, thereby offering new insights for the clinical management of this condition.

## 1 INTRODUCTION

Oral squamous cell carcinoma (OSCC) is a prevalent type of cancer that arises from the mucosal epithelium of the oral cavity, with a high prevalence rate accounting for an estimated 377,713 new cases worldwide annually [1, 2]. The latest global estimates show that in 2018, there were 177,384 deaths from oral cancer. Traditional risk factors for oral cancer include smoking and heavy alcohol consumption. Staging and grading of OSCC are essential for management as they impact risk stratification and are the first step in achieving personalized treatment [3-6]. Due to the lack of specific clinical manifestations in the early stage of the disease, nearly 40% of the patients were found in the late stage of OSCC, and more than half of the patients relapsed after surgical treatment [7]. At present, the main treatment method for OSCC is a comprehensive sequence therapy, including radiotherapy, chemotherapy, and gene therapy on the basis of surgical resection of the tumor, but the therapeutic effect is not satisfactory. Overall, the survival rate and prognosis of OSCC are unsatisfactory. Therefore, it is urgent to find a new therapeutic strategy with low-risk and effective agents to solve this problem [8-10].

The activation of the NFκB pathway plays a significant role in various tumors, including OSCC. It is intricately involved in the initiation and progression of tumors by extensively modulating gene transcription and contributing to tumor growth, .angiogenesis, anti-apoptosis, invasion, and metastasis [11, 12]. There are five members in the NFκB family of transcription factors: NFκB1 (p50/p105), NFκB2 (p52/p100), c-Rel, RelA (p65), and RelB [13, 14]. Two major signaling pathways governed by NFκB are crucially involved: classical pathways and non-classical pathways. The classical pathway is triggered by cytokines like tumor necrosis factor TNF-α, interleukin (IL)-1β, lipopolysaccharide (LPS), and receptor activator of NFκB ligand (RANKL), leading to IκB kinase (IKK) activation, phosphorylation of IκB by IKK2, and degradation of IκB, which releases the p50/P65 dimer, which is then translocated into the nucleus and involved in regulating the expression of genes important in cell proliferation, survival and DNA damage repair. In the non-classical pathway, upstream factors activate NFκB-inducible kinase (NIK). NIK, in turn, phosphorylates IKK1, resulting in phosphorylation of p100. Subsequent ubiquitination and degradation of p100 enables the transport of the RelB-p52 dimer into the nucleus [15, 16].

IKK2 is an important kinase in the activation of the NFκB classical pathway, and inhibition of its activity can reduce the activation of the NFκB pathway. LY2409881 is a potent and selective inhibitor of IKK2 [17]. The current study shows that in diffuse large B- cell lymphoma (DLBCL) cells, LY2409881 inhibits the activated NFκB pathway, causing concentration- and time-dependent cell growth inhibition and apoptosis [18]. In SUDHL22 and LY1 cells, LY2409881 is synergized with the histone deacetylase (HDAC) inhibitor romidepsin to inhibit cell growth [19].

MLN4924, a selective and potent small-molecule inhibitor of the NEDD8-activating enzyme, has demonstrated its anti-tumor efficacy across various malignant tumor types [20, 21]. By forming a covalent bond with NEDD8 molecules, MLN4924 disrupts the neddylation modification process, leading to the inhibition of the NEDD8-MLN4924 complex formation. This effectively blocks the polyubiquitination and subsequent degradation of a range of intracellular proteins regulated by CRLs, which are crucial for cell cycle progression and signal transduction. Therefore, MLN4924 exerts its anti-tumor effects by preventing the degradation of multiple CRL substrates [22, 23]. Soucy *et al.* [24] first reported the antitumor effect of MLN4924. They found that in the colorectal cancer cell line HCT116, MLN4924 could promote apoptosis by regulating DNA synthesis in the S phase of cells. Chen *et al.* [25] found that in acute myeloid leukemia (AML), MLN4924 inhibited cell proliferation, promoted apoptosis, and induced cell cycle arrest in the G2/M phase. Since MLN4924 can exert anti-tumor effects by extensively affecting the cell cycle and promoting apoptosis, it has entered a phase I clinical trial. In the NFκB pathway, polyubiquitination and degradation of IκB proteins can be mediated by CRL1βTRCP, which is tightly regulated by NAE. Therefore, we believe that MLN4924 may be a new therapeutic strategy for OSCC by inhibiting the ubiquitination of various proteins and inhibiting the NFκB pathway.

This study aimed to evaluate the impact of LY2409881 and MLN4924, two inhibitors targeting NFκB, on the susceptibility of OSCC cell lines CAL27 and SCC15 to apoptosis and their proliferation rate. Furthermore, the findings were substantiated by conducting *in vivo* tumorigenic experiments. LY2409881 may be a new drug for the treatment of oral squamous cell carcinoma, providing a new approach to the clinical treatment of oral squamous cell carcinoma. MLN4924 may exert its anti-oral squamous cell carcinoma effect by inhibiting the NFκB pathway and increasing the expression of multiple anti-cancer genes, demonstrating the potential for the treatment of oral squamous cell carcinoma.

## 2 MATERIALS AND METHODS

### 2.1 Cell Lines and Culture

The CAL27 and SCC15 cell lines, derived from OSCC, were generously provided by Professor Wantao Chen from the Shanghai Institute of Stomatology and the Key Laboratory of Stomatology Biomedicine at Wuhan University. The cells were cultured in a 37°C cell incubator with a 5% CO_2_ atmosphere, using high glucose (DMEM) medium supplemented with 10% fetal bovine serum (Gibco, New York, NY, USA) and 1% penicillin/streptomycin (P/S; containing penicillin 100 U/mL and streptomycin 100 µg/mL; Thermo Fisher). These cell lines have been extensively utilized in numerous studies, particularly in investigations pertaining to the cytotoxicity of antitumor drugs.

### 2.2 MTT Cell Viability Assay

A total of 5×103 cells were seeded per well in 96-well plates. The cells were then treated with MLN4924 at various concentrations (control group, 0.01 μM, 0.03 μM, 0.1 μM, 0.3 μM, 1 μM, 3 μM) and LY2409881 at different concentrations (control group, 5 μM, 10 μM, 15 μM, 20 μM, 25 μM, 30 μM). Following treatment, the medium was replaced after 24, 48, and 72 hours, respectively. Subsequently, each well was supplemented with 300μL of 3-(4,5-dimethylthiazol-2-yl) -2,5-diphenyltetrazolium bromide (MTT, Sigma-Aldrich, St. Louis, MO, USA) solution at a final concentration of 0.5mg/mL. The cells were incubated for 4 hours, and the absorbance at 570 nm was measured using a microplate reader (Bio-Rad, Hercules, CA, USA). The IC_50_ values, indicating the 50% inhibitory concentration, were calculated using GraphPad Prism software (Version 8.0, San Diego, CA, USA).

### 2.3 Analysis of Cell Apoptosis by Flow Cytometry

To evaluate the level of apoptosis, an apoptosis detection kit (Annexin V-FITC/PI; Biolegend, San Diego, CA, USA) was employed. The cells were cultured in either untreated media or media treated with a stimulator. After harvesting, the cells were suspended in 1x binding buffer and stained with Annexin V-FITC and PI as per the manufacturer's protocol. Subsequently, the cell samples were analyzed within an hour using a FACS flow cytometer (BD Biosciences).

### 2.4 Cell Cycle Detection

Following a 24-hour period of serum starvation, the cells were washed with PBS and subsequently collected. Subsequently, the cells were fixed with 70% ethanol and stained with propidium iodide (PI). The resulting cell samples were then analyzed using the FACS Calibur system (BD Biosciences, San Jose, CA, USA).

### 2.5 Western Blotting Analysis

Cells were collected at 80%-90% confluency. RIPA lysis buffer and a protease inhibitor cocktail (Sigma-Aldrich, Saint Louis, MO, USA) were used to extract total cellular proteins. Protein samples (30-40 ug) were separated on a 6% or 8% polyacrylamide gel and transferred onto a PVDF membrane (Immobilon-P, USA) with a pore size of 0.45 μm. The antigen was blocked with 4% BSA for 12 hours at 4°C. Subsequently, the membranes were treated with a primary antibody, followed by incubation with horseradish peroxidase (HRP)-conjugated secondary antibody (Bioss, China; 1:10000), the primary antibody included Bcl-2 antibody(WANLEIBIO, China, WL01556; 1:700), anti-IKK1(WANLEIBIO, China, WL00053; 1:300), anti-IKK2(WANLEIBIO, China, WL04340; 1:300), anti-NF-κB p50(WANLEIBIO, China, WL01917; 1:500), anti-Cleaved-casp3(WANLEIBIO, China, WL02117; 1:500), anti -NF-κB p65(WANLEIBIO, China, WL01273b; 1:1000), anti-NF-κB p21 (WANLEIBIO, China, WL0362; 1:500), anti-CyclinD1(WANLEIBIO, China, WL01435a; 1:500), RORα(Abcam, UK, EPR23719-18; 1:1000), and Bmal1(Abcam, UK, EPR20906-14; 1:1000). The enhanced chemiluminescence detection kit reagent was used to detect the bands. β-actin(WANLEIBIO, China, WL01372; 1:500) was used as a loading control throughout.

### 2.6 Xenograft Tumor Model

The Institutional Review Board (IRB) of Central South University's Hunan Xiangya Stomatological Hospital approved the research protocol under reference number 20200002. Further, 4-week-old BALB/C nude mice were purchased from Shanghai Slack Laboratory Animal Company and raised in the SPF Laboratory Animal Center of the State Key Laboratory of Medical Genetics, Central South University. Each 100 μL CAL27 cell solution was subcutaneously injected into nude mice near the axilla. Before the first administration, a vernier caliper was used to measure the shortest diameter, longest diameter, and body weight of the tumor once after each administration cycle, and the tumor volume was calculated according to the formula: tumor volume = 1/2 (long diameter × short diameter 2). Mice were anesthetized with 25 mg/kg of Zoletil 50 and 10 mg/kg of Rompun by intraperitoneal injection. We confirm that all methods were conducted in accordance with relevant guidelines and regulations, and all methods are reported in accordance with ARRIVE guidelines.

### 2.7 Tunel Staining

The sections were routinely dehydrated and covered with proteinase K working solution dropwise to permeate the tissue. Incubation was carried out at 37°C for 15 minutes, followed by the addition of an adequate amount of membrane-breaking working solution to cover the tissue. After incubating at room temperature for 10 minutes and allowing the sections to dry slightly, each section was treated with an appropriate amount of reagent 3 (converter-POD). The tissue was covered, washed with PBS, DAB chromogenic solution was added, counterstained with hematoxylin, and mounted with gum. Brown staining showed apoptotic cells and blue staining showed normal living cells.

### 2.8 Statistical Analysis

For analysis in this project, GraphPad Prism 6.0 software was utilized. The data are expressed as either mean ± SD or SEM. To compare multiple groups with homogeneous variances, one-way ANOVA followed by Tukey's multiple comparison test or unpaired t-test was employed. In cases where variances were unequal, the rank sum test with Welch's correction was applied. A significance level of *p* < 0.05 was considered statistically significant and denoted by *, highly significant *p*-values (<0.01) indicated by **, and very significant *p*-values (<0.001) denoted by ***.

## 3 RESULTS

### 3.1 NFκB Inhibitors Inhibit the Proliferation of CAL27 and SCC15 Cells

The effects of LY2409881 and MLN4924 on the proliferation of CAL27 and SCC15 cells were detected by MTT cell proliferation assay *in vitro*. Figs. (**1A**-**D**) summarizes the results of each inhibitor on cells. The inhibitory effects of the two inhibitors on the proliferation of Cal27 and SCC15 cells were both concentration- and time-dependent. In terms of IC_50_, CAL27, and SCC15 cell proliferation inhibition, both inhibitors were more effective on the CAL27 cell line. After LY2409881 treatment of CAL27, the IC_50_ value of 24 to 72 h was between 29.22-8.87 μM. After SCC15 cell treatment, the IC_50_ value was between 40.8 μM and 19.8 μM for 24-72 h. After CAL27 was treated with MLN4924, the IC_50_ value was between 8.1-0.62 μM at 24-72 h, and that of SCC15 cells was between 25-1.5 μM at 24-72 h. MLN4924 was more effective than LY2409881.

### 3.2 LY2409881 and MLN4924 Induce the Apoptosis of CAL27 and SCC15 Cells

**Fig. (2):**
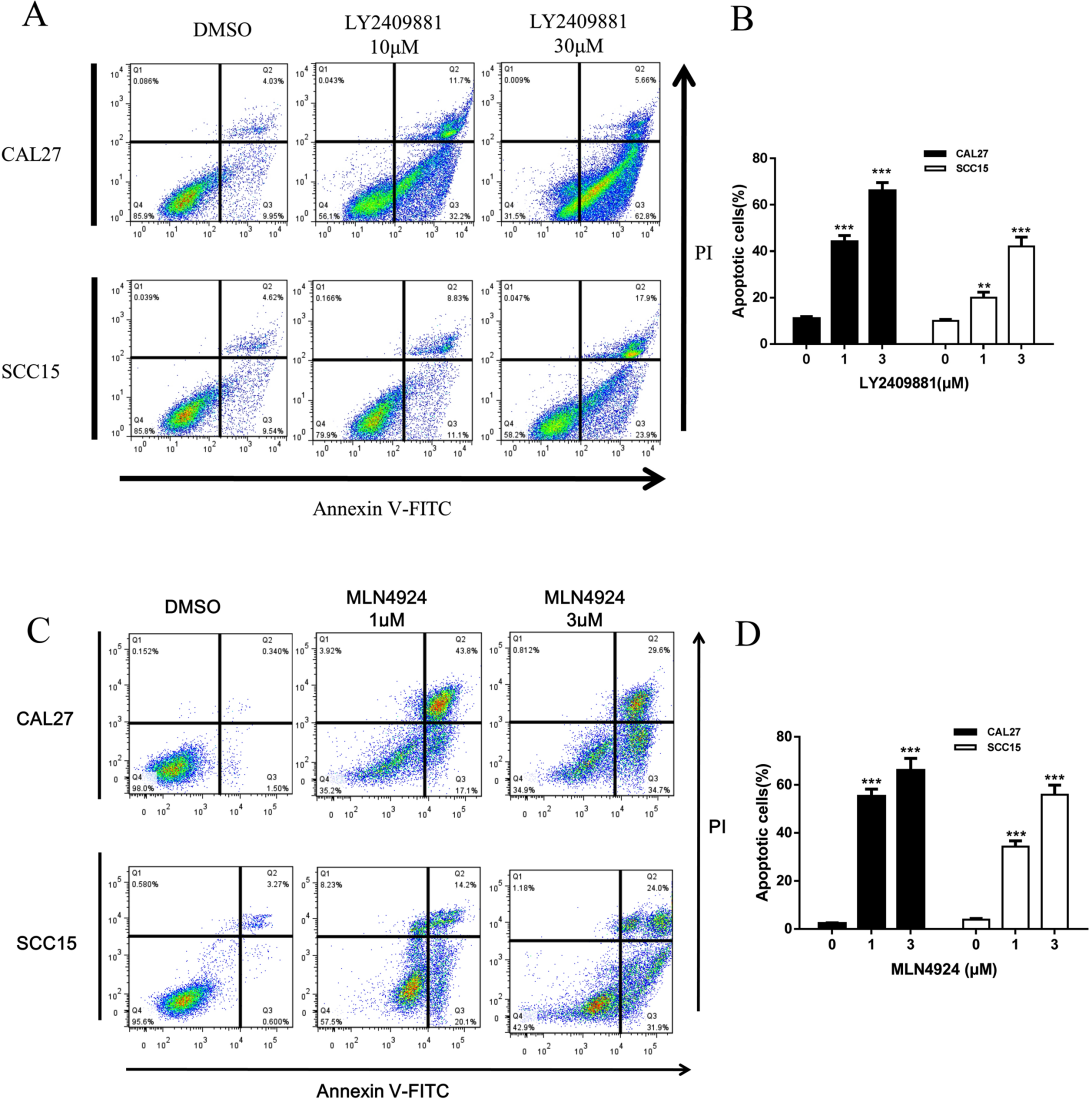
(**A**-**D**) The inhibitor could induce apoptosis in CAL27 and SCC15 cells. The CAL27 and SCC15 cell lines were seeded into 6-well plates, followed by treatment with either DMSO in the control group or LY2409881 or MLN4924 in the experimental group for 48 hours. The detection of apoptotic cells was performed using Annexin V-FITC/PI double staining. Each group included 2 duplicate samples, and the experiments were independently repeated three times. The results are presented as the mean ± standard error of the mean (SEM). Statistical significance is denoted as ** *p* < 0.01, *** *p* < 0.001, **** *p* < 0.001.

To investigate the underlying mechanism of the anti-proliferation effect of these inhibitors on OSCC cells, we checked the apoptosis in two cell lines using PI/AnexinV staining. The data showed that compared with the control group, the proportion of apoptosis cells was significantly increased in the group treated with LY2409881 or MLN4924 for 48 h (Figs. **2A**-**D**). Both cells underwent concentration-dependent apoptosis in the presence of inhibitor treatment. The effects of the LY2409881 inhibitor on apoptosis were as follows: 10 μM LY2409881 was greater than DMSO, 30μM LY2409881 was greater than DMSO, 30 μM LY2409881 was greater than 10 μM LY2409881 (compare the number of apoptotic cells), and CAL27 cells had more apoptosis and apoptosis was evident in SCC15 cells. The effects of MLN4924 inhibitor on apoptosis were as follows: 1 μM was greater than DMSO, 3 μM was greater than DMSO, 3 μM was greater than 1 μM, (compare the number of apoptotic cells, ***p<0.001;), and CAL27 cells treated with the same concentration Apoptosis was more obvious than that of SCC15 cells (*p*<0.01).

### 3.3 Mechanism of LY2409881 Promoting Apoptosis in CAL27 Cells

To explore the inhibitory mechanism of LY2409881 on the proliferation of CAL27 cells, we investigated the expression of the B-cell lymphoma-2 (Bcl-2) protein and its related apoptosis pathway. CAL27 cells were exposed to different concentrations of LY2409881 (0 μM, 10 μM, 30 μM) for 48 hours, and the findings are depicted in Figs. (**3A**, **B**). As the concentration of LY2409881 treatment increased, there was a gradual decrease in the expression of the anti-apoptotic protein Bcl-2. Concurrently, the expression of NFκB pathway proteins, including IKK2, IKK1, P50, and p65, was reduced upon treatment with LY2409881. Notably, LY2409881 exhibited stronger selective inhibition of IKK2 compared to IKK1(*p*<0.05).

### 3.4 MLN4924 Induces Apoptosis of CAL27 and SCC15 Cells, and its Mechanism

CAL27 and SCC15 cell lines were exposed to MLN4924 (0.3 μM, 1 μM) for a duration of 48 hours alongside a control group treated with 1% DMSO simultaneously. Fig. (**4A**) illustrates the cell cycle distribution numbers of CAL27 cells in the G2 phase, which were 50.00% ± 2.00 and 62.03% ± 2.23 for the two MLN4924 concentrations, respectively. Similarly, the cell cycle distribution numbers of SCC15 cells in the G2 phase were observed to be 30.14% ± 2.56 and 62.03% ± 3.22, respectively (***p<0.001). Notably, all the cell lines displayed an accumulation in the G2/M phase, with a significant increase in the number of quadruple cells. This indicates cell cycle arrest at G2/M, subsequently inhibiting cell proliferation.

In order to explore the mechanism of MLN4924 inhibitor on apoptosis of CAL27 and SCC15 cells, the protein expressions of p21 and CyclinD1 in the relevant cell cycle were detected, and the control group was 0μM (containing DMSO, 1%). The OSCC cell line was treated with MLN4924 (0.3 μM, 1 μM) in the experimental group for 48 h. As shown in Fig. (**4B**), the expression of p21 protein in CAL27 and SCC15 cells increased with the increase of MLN4924 concentration; CyclinD1 protein expression was significantly decreased in the administration group (*p*<0.05). The expression of related apoptotic proteins is shown in Fig. (**4C**). Cleaved-casp3 protein in CAL27 and SCC15 cells increased with the increase of MLN4924 concentration; the expression of Bcl-2 protein in the drug-added group was significantly decreased. The expressions of NFκB pathway protein-related proteins IKK1/IKK2 and pIκBα are shown in Fig. (**4D**). The protein expression of IκBα increased with the increase of MLN4924 concentration; the protein expression of IKK1/2 in the drug-added group was significantly decreased (*p*<0.05).

### 3.5 Inhibitory Effect of the Inhibitor on Tumorigenesis of CAL27 Cells Transplanted in Nude Mice

**Fig. (5):**
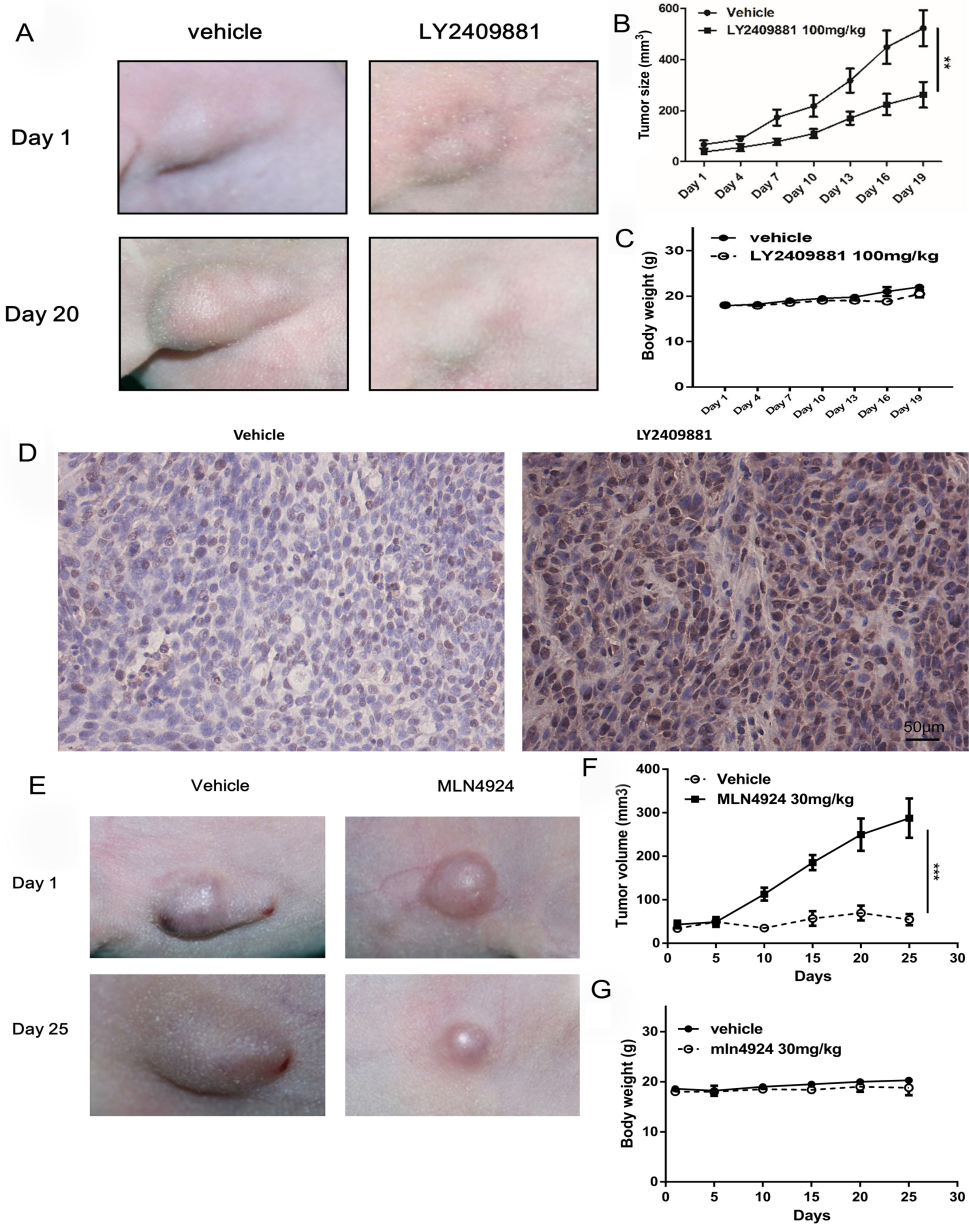
LY2409881 and MLN4924 have inhibitory effects on tumorigenesis of CAL27 cells xenografted in nude mice. (**A**) Nude mice were inoculated with CAL27, injected with vehicle and LY2409881, and compared 20 days later. (**B**) The tumor volume of nude mice was measured every three days, tumor volume = 1/2 (long diameter × short diameter); (**C**) The change of nude mice body weight every three days. (**D**) Tunel staining and brown staining showed apoptotic cells; blue staining showed normal living cells, and the number of apoptotic cells in the LY2409881 treatment group was significantly higher than that in the control group; (**E**) Nude mice were inoculated with CAL27 and injected with vehicle and MLN4924, and compared 25days later; (**F**) The tumor volume of nude mice at an interval of 5 days; (**G**) Changes in the body weight of nude mice. Data are presented as mean ± SEM (n=5 in the control group, n=5 in the administration group), and data were analyzed using one-way ANOVA, ** *p* < 0.01;*** *p* < 0.001.

In this study, a nude mouse xenograft tumor model was established using CAL27 cells to investigate the inhibitory effect of inhibitors on OSCC *in vivo*. The dorsal side of 4-week-old BALB/C nude mice near the axilla was injected subcutaneously with CAL27 cells at a density of 2x106/100 μl. The tumor formation rate in this experiment was 100% (the control group n=5, the experimental group n=5). Each time, a dosage of 100 mg/kg body weight was administered, and no deaths occurred during the treatment period in the mice. Figs. (**5A**, **B**) demonstrates that the LY2409881 inhibition group exhibited a significantly smaller tumor volume compared to the control group, while the body weight of the two groups of nude mice did not show significant changes (Fig. **5C**, *p*<0.05). Fig. (**5D**) demonstrates the results of TUNEL staining, which revealed a higher positive rate of TUNEL (brown staining) in tumors formed by nude mice in the LY2409881 experimental group compared to the control group (Vehicle) (*p*<0.05). This indicates that the LY2409881 administration group exhibited significantly higher tumor cell apoptosis than the control group. Similarly, Figs. (**5E**, **F**) shows that the tumor volume of the MLN4924 inhibition group was significantly smaller than that of the control group, and there were no significant changes in the body weight of the nude mice in both groups, as depicted in Fig. (**5G**) (***, *p*<0.001). The inhibitory effects on tumorigenesis were consistent for both inhibitors, with MLN4924 proving to be more effective than LY2409881.

## 4 DISCUSSION

The significance of inflammation in cancer has long been recognized, and it is now further amplified with the emergence of NFκB inhibitors. This pathway is crucial in inflammation and presents a promising target for cancer treatment [2]. NFκB, discovered in 1986, is often activated in cancer and promotes cell proliferation and survival, making it a long-standing target for cancer research [26-30]. The discovery of potent and selective inhibitors targeting NFκB has historically focused on IKK2 inhibitors [31]. Although dozens of IKK2 inhibitors have been reported, none have entered the clinic.

As the 11^th^ most prevalent cancer globally [32, 33], oral cancer imposes a significant burden on public health and mortality. Presently, the primary approach to treating OSCC involves surgical resection combined with adjuvant or neoadjuvant chemoradiotherapy [34, 35]. However, despite these efforts, approximately 60% of patients experience local recurrence, while 30% develop distant metastases [36]. The lack of available targeted therapies that effectively address OSCC [37, 38] underscores the urgent need for innovative treatment modalities to enhance the clinical outcomes associated with this condition.

Given the well-established association between OSCC and the NFκB pathway, the utilization of NFκB inhibitors holds particular promise for addressing this disease. In our study, we employed two inhibitors, LY2409881 and MLN4924, to target distinct nodes within the NFκB signaling pathway. Our aim was to investigate the mechanisms through which these inhibitors effectively hinder cell proliferation, promote apoptosis, and inhibit NFκB pathway-mediated apoptosis in two OSCC cell lines.

In this study, it was observed that MLN4924 exhibited a more pronounced inhibitory effect on cell proliferation compared to LY2409881. Specifically, the IC_50_ values of MLN4924 for CAL27 and SCC15 cells after 48 hours were found to be 0.87 μM and 3.3 μM, respectively. This implies that MLN4924 demonstrated a lower IC_50_ value within the tested dose range. At the same time, it should be noted that these concentrations may still be relatively high for the agents to be utilized solely as antiproliferative drugs; the observed antiproliferative effect can be regarded as an additional favorable outcome.

The promoting effect of NFκB pathway inhibitor on apoptosis was also confirmed. Importantly, this effect was observed in both cell lines studied. Apoptosis is the result of the comprehensive action of internal and external factors, and its process is precisely regulated by apoptotic genes [39, 40]. Bcl-2 gene is one of the most important anti-apoptotic oncogenes [41], and Cleaved-casp3 is the main executor of apoptosis [42, 43]. Our results showed that in the CAL27 cell line, the expression of anti-apoptotic protein Bcl-2 was gradually decreased with increasing concentrations of LY2409881 and MLN4924. Meanwhile, the expression of Cleaved-casp3 in the SCC15 cell line gradually increased with the increase of MLN4924 concentration. This indicates that both LY2409881 and MLN4924 may induce apoptosis by inhibiting the expression of Bcl-2, and the promotion of apoptosis by MLN4924 is also related to the activation of Cleaved- casp3.

In the classical pathway of NFκB activation, stimulation of various signals leads to the activation of IKK2. IKK2 phosphorylates IκB, which polyubiquitinates and degrades IκB. LY2409881 is designed to be a potent inhibitor of IKK2 in the NFκB pathway [44]. As an inhibitor of NAE, MLN4924 can inhibit the activity of CRLs, increase the concentration of IκB protein, and reduce the activity of NFκB [45, 46]. Our results showed that both inhibitors reduced the expression of NFκB pathway proteins.

In our *in vitro* experiments, we observed that LY2409881 and MLN4924 exhibited inhibitory effects on the growth of oral squamous cell carcinoma CAL27 and SCC15 cells. However, CAL27 cells displayed greater sensitivity to these inhibitors compared to SCC15 cells, leading to an increase in apoptosis. In this study, we selected nude mice xenografted CAL27 cells and transplanted them by subcutaneous inoculation. After the tumor-bearing nude mice were continuously treated with LY2409881 and MLN4924, although the tumor volume still increased, the growth rate of the inhibitor treatment was significantly slower than that of the control group, and the inhibitor had no significant effect on the body weight of the mice in the inhibitor group and the control group, which means that LY2409881 and MLN4924 were well tolerated *in vivo*. Our study shows that LY2409881 and MLN4924 can inhibit the growth of OSCC CAL27 cell xenografts in nude mice.

Our findings from the experiments indicate that both LY2409881 and MLN4924 inhibitors possess the potential to induce apoptosis in CAL27 and SCC15 cells. The underlying mechanism could involve the inhibition of the NFκB pathway. Furthermore, our data suggests that MLN4924 exhibits greater suitability for clinical applications. It is imperative to conduct further *in vivo* investigations to ascertain the efficacy of this inhibitor in treating OSCC.

In our *in vitro* studies, LY2409881 and MLN4924 demonstrated significant inhibition of the proliferation of the CAL27 and SCC15 oral squamous cell carcinoma cell lines. Additionally, these inhibitors effectively induced apoptosis in both CAL27 and SCC15 cells. Furthermore, they exhibited promising potential in preventing the growth of CAL27 cell xenografts in nude mice. Notably, these inhibitory effects may be mediated through the classical activation pathway of NFκB. Importantly, MLN4924 showed superior efficacy in inhibiting cell proliferation and promoting apoptosis compared to LY2409881. These findings highlight the potential of LY2409881 and MLN4924 as novel therapeutic agents for OSCC, thereby offering new insights for the clinical management of this condition.

## CONCLUSION

Of course, our study also has many limitations. We still have gaps in our research on LY2409881, and we have not explored the specific regulatory mechanisms and downstream targets of the two drugs in OSCC, which is an area we will strive to address in the future. Furthermore, the specific role of NFκB in the immune microenvironment of OSCC should also be a focus for future scholars to explore.

## Figures and Tables

**Fig. (1):**
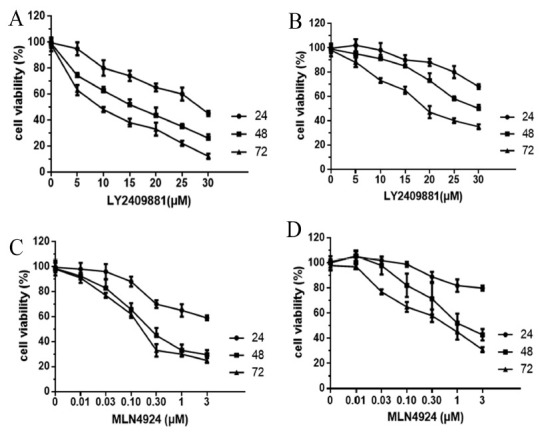
(**A**-**D**) Inhibition of proliferation by LY2409881 and MLN4924 in OSCC cell lines. The cells were stimulated with LY2409881 and MLN4924 at various concentrations (indicated) for 24, 48, and 72 hours. Each concentration was tested in six replicate wells. The mean values of these experiments were determined at three independent time points (n=3).

**Fig. (3):**
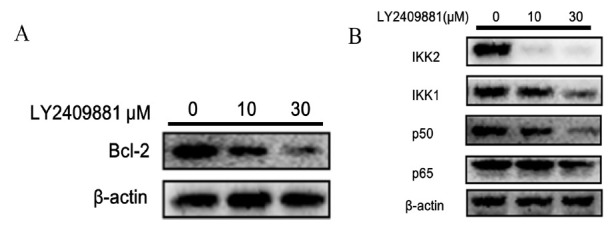
Mechanism of LY2409881 promoting apoptosis in CAL27 cells. To assess the impact of varying concentrations of LY2409881 (0 μM, 10 μM, and 30 μM) on CAL27 cells, Western blot analysis was employed to analyze the expression levels of relevant proteins after 48 hours of treatment. The following protein expressions were examined: (**A**) Bcl-2, a protein associated with tumor apoptosis, and (**B**) Proteins involved in the NFκB pathway, including IKK2, IKK1, p50, and p65. The internal reference for normalization was β-actin. The experiments were independently repeated three times to ensure reliability.

**Fig. (4):**
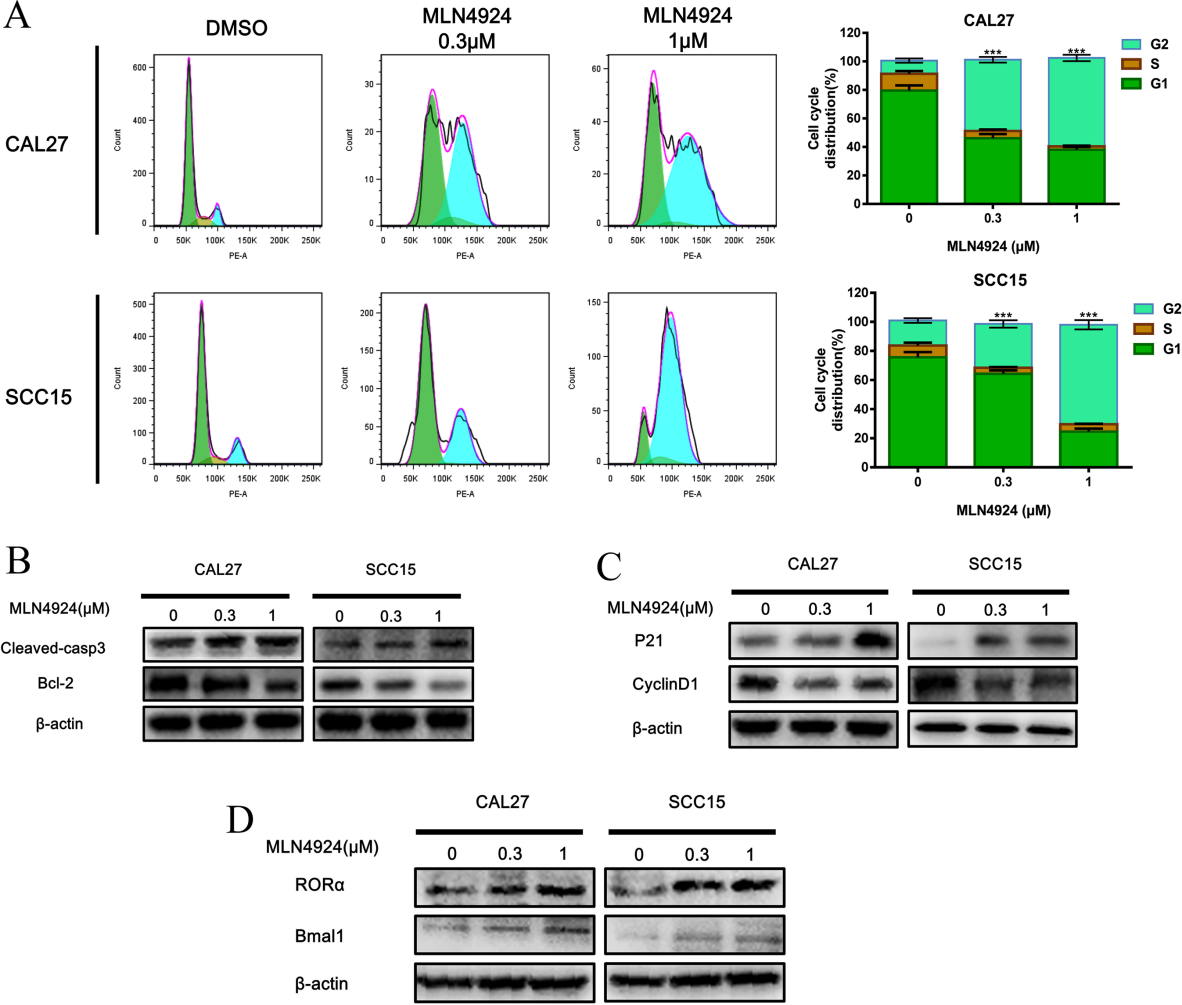
Mechanisms of MLN4924 promote apoptosis in CAL27 and SCC15 cells. (**A**) CAL27 and SCC15 cells were divided into 6-well plates and then treated with MLN4924 (0 μM, 0.3 μM, 1 μM) for 48 h. DNA copy number was detected by flow cytometry, and the cell cycle was arrested in the G2/M phase; (**B**) Control group 0 μM (containing DMSO, 1%) and MLN4924 treated with two different concentrations (0.3 μM, 1 μM) for 48 h, and expression of related apoptosis proteins Cleaved-caspase3 and Bcl-2 by western blot; (**C**) Protein expression of cell cycle p21 and CyclinD1; (**D**) Protein expression of anti-tumor markers Bmal1 and RORα, *** *p* < 0.001.

## Data Availability

The data and supportive information are available within the article.

## LIST OF ABBREVIATIONS

OSCC: Oral Squamous Cell Carcinoma
IL: Interleukin
LPS: Lipopolysaccharide
IKK: Leading to IκB Kinase
NIK: NFκB-inducible Kinase
DLBCL: Diffuse Large B-cell Lymphoma
AML: Acute Myeloid Leukemia
PI: Propidium Iodide
